# Linking machine learning and biophysical structural features in drug discovery

**DOI:** 10.3389/fmolb.2024.1305272

**Published:** 2025-01-23

**Authors:** Armin Ahmadi, Shivangi Gupta, Vineetha Menon, Jerome Baudry

**Affiliations:** ^1^ Department of Biological Sciences, The University of Alabama in Huntsville, Huntsville, AL, United States; ^2^ Department of Computer Science, The University of Alabama in Huntsville, Huntsville, AL, United States

**Keywords:** drug discovery, machine learning, pharmacophore, conformational selection, docking, ensemble docking, chemical biology

## Abstract

**Introduction:**

Machine learning methods were applied to analyze pharmacophore features derived from four protein-binding sites, aiming to identify key features associated with ligand-specific protein conformations.

**Methods:**

Using molecular dynamics simulations, we generated an ensemble of protein conformations to capture the dynamic nature of their binding sites. By leveraging pharmacophore descriptors, the AI/ML framework prioritized features uniquely associated with ligand-selected conformations, enabling a mechanism-driven understanding of binding interactions. This novel approach integrates biophysical insights with machine learning, focusing on pharmacophoric properties such as charge, hydrogen bonding, hydrophobicity, and aromaticity.

**Results:**

Results showed significant enrichment of true positive ligands—improving database enrichment by up to 54-fold compared to random selection—demonstrating the robustness of this approach across diverse proteins.

**Conclusion:**

Unlike conventional structure-based or ligand-based screening methods, this work emphasizes the role of specific protein conformations in driving ligand binding, making the process highly interpretable and actionable for drug discovery. The key innovation lies in identifying pharmacophore features tied to conformations selected by ligands, offering a predictive framework for optimizing drug candidates. This study illustrates the potential of combining ML and pharmacophoric analysis to develop intuitive and mechanism-driven tools for lead optimization and rational drug design.

## 1 Introduction

Contemporary approaches in computational drug discovery and computational chemical biology are mostly centered around protein: ligand predictions and rationalizations, in particular through using docking approaches. Docking is not only used to identify a specific binding mode for a particular ligand in a given protein target, but it is also used, and maybe mostly so nowadays, as a virtual screening tool that will “reduce the size of the chemical haystack” and allow a faster and more economical identification of drug candidate “needles.” There are many approaches, tools, and scales of virtual screening through docking. Our group and the groups of several colleagues and collaborators have experienced success over the last few years in ligand discovery by using more than one structure for the protein targets (Evangelista et al., 2016; Amaro et al., 2018; Evangelista et al., 2019). By success, we mean that the enrichment of a database in actual ligands after docking is significantly increased compared to the enrichment of the database in ligands prior to docking. We and our collaborators have achieved these results by modeling a conformational selection mechanism rather than an induced fit mechanism for ligand binding. In conformational selection, the ligands “select” specific protein conformations to form a protein–ligand combination that is thermodynamically favorable. The key to a successful docking campaign is as follows: like other groups, we mostly have used an ensemble docking method, where multiple protein conformations are generated using MD and used for in multiple-protein conformation docking calculations.

As specified above, this approach has not only proven valuable to vastly increase database enrichment by identifying in retrospect, from known ligands: decoys database, these protein conformations that are indeed massively selected by the proteins’ ligand. Over the last few years, our collaboration has used machine learning approaches to identify the properties that such specific protein conformations may possess that are associated with their selection by ligands (Akondi et al., 2019; Sripriya Akondi et al., 2022; Gupta et al., 2022a; Gupta et al. 2022b; Gupta et al. 2023). This was successful, but the approach used combination protein descriptors that are quite general and, not unlike the case of QSAR for ligand-based discovery, not necessarily easily explainable in terms of physical properties of the protein target’s selected conformations (Gupta et al., 2023).

As stated by the editors in the description of this special issue of *Frontiers in Molecular Biosciences*, there is a need for “*research that utilizes ML protocols/architectures but offers a detailed and comprehensive interpretation of observed phenomena*.” This work is the first step toward applying sophisticated machine learning approaches but, instead of processing arcane and obscure protein descriptors occasionally, we have used pharmacophoric descriptions of the protein target’s binding sites in this study (Śledź and Caflisch, 2018). This has the advantage of allowing a direct and straightforward rationalization of the binding site’s features associated with conformation selection, in terms of charges, hydrogen bond potential, hydrophobicity/hydrophilicity, and resonance of the protein environment, which are indeed concepts used to rationalize binding and optimize hits and leads (Leach et al., 2009).

## 2 Methods

The four target proteins that were used in the present study as extensive molecular dynamics trajectories and characterization of the conformation, leading to ligand binding, are available (Evangelista et al., 2019; Gupta et al., 2022a; Gupta et al., 2023).

### 2.1 Molecular dynamics

In this study, we used the molecular dynamics conformations obtained from MD simulations of a previous work in our laboratory, involving our four GPCR structures of interest [adenosine receptor A2A, β2-adrenergic receptor, δ-type opioid receptor, and κ-type opioid receptor, as listed in Table 1] (Evangelista et al., 2019). These structures were downloaded from the RCSB Protein Data Bank (RCSB-PDB) and were optimized by deleting non-native domains and co-crystallized ligands, and building missing loops. The proteins were then placed in a bilayer membrane with a lipid composition of phosphatidylcholine (42%), phosphatidylethanolamine (25%), phosphatidylserine (14%), and cholesterol (19%). Coarse-grained models were used to reduce the systems from approximately 125,000 full atoms to around 14,000 CG particles. Gromacs v5.1.0 software was used for 600-ns MD simulations, and frames were saved every 200 ps, representing 3,000 conformations for each protein. These simulations ran on the MolDyn High-Performance cluster at the UT/ORNL Center for Molecular Biophysics, Oak Ridge, Tennessee, provided the structural basis for our pharmacophore and machine learning analysis, offering insights into the dynamic nature of these proteins’ binding sites. In the current study, we used a set of 3,000 MD conformations for each of the proteins (i.e., 12,000 structures in total for the four proteins used here).

**TABLE 1:** Target proteins and the PDB crystal structures used in this study.

Protein name	Gene name	PDB ID	Reference
Adenosine receptor A2A	*ADORA2A*	3EML	Jaakola et al. (2008)
β2-Adrenergic receptor	*ADRB2*	2RH1	Cherezov et al. (2007)
δ-Type opioid receptor	*OPRD1*	4N6H	Fenalti et al. (2014)
κ-Type opioid receptor	*OPRK1*	4DJH	Wu et al. (2012)

### 2.2 Conformation preparation

All molecular dynamics conformations were imported in a MOE database (*Molecular Operating Environment*, Chemical Computing Group Ltd., Montreal, Canada, 2022). These conformations were then superposed on the first MD frame based on heavy atoms of the pocket residues. Atomic partial charges were assigned from the MMFF94x force field, as implemented in MOE.

### 2.3 Pharmacophore generation

The *SiteFinder* facility from MOE was used to identify potential active sites in the first conformation of the molecular dynamics trajectories with a focus on the known ligand-binding sites to concentrate on the most functionally significant areas of the proteins. SiteFinder is based on the concept of alpha shapes, which represent a more generalized form of convex hulls (Edelsbrunner and Mücke, 1994).

Pharmacophore feature generation was conducted using the *DB-PH4* facility in MOE within a 6.5-Å cutoff from the SiteFinder binding site using the “unified scheme” pharmacophore definitions in MOE: hydrogen bond donor (Don); hydrogen bond acceptor (Acc); cation (Cat); anion (Ani); aromatic center or non-aromatic π-system ring, in which each atom is sp2 hybridized (Aro); and hydrophobic atoms and hydrophobic centers (Hyd).

The default pharmacophore radii sizes are set at 1.2 Å for Acc and Don features, 1.4 Å for aromatic centers, and 1.6 Å for hydrophobic features. Hydrophobic features that are within 1 Å to one another are clustered into a single feature with an increased radius, up to a maximum size of 3 Å. A Boolean “and” is defined for overlapping of “Don & Cat” or “Don & Acc” features. Finally, using the *database autoPH4* facility in MOE, the pharmacophore features extracted from all protein MD trajectories are clustered together to create consensus features. These consensus features reflect the frequency with which specific pharmacophore features occur in a particular region of space in the database.

### 2.4 AI/ML feature ranking framework

The pharmacophores generated were first translated into a binary encoded database by utilizing the available frequency data. Later, it was then subjected to an AI/ML feature ranking framework to identify and choose distinctive pharmacophoric characteristics for each protein. The process of feature selection for developing a predictive model entails reducing the number of input variables. In some cases, limiting the amount of input variables may improve model performance while also cutting modeling computing costs.

The key approach here is to identify the pharmacophore features that are specifically associated with the protein conformations selected by the ligands. These selected conformations were identified and described in our previous work (Evangelista et al., 2019) and used in our previous publications (Gupta et al., 2022a; Gupta et al., 2023) to identify global protein properties correlated with ligand binding. Here, as described above, we are attempting to identify pharmacophore features associated with ligand binding rather than global—and often obscure—protein descriptors.

To do so, we used four different ML feature selection algorithms to identify the key pharmacophore properties and eliminate the redundant properties to maximize the prediction of probable protein-binding conformations while reducing dataset complexity. Analysis of variance (ANOVA), mutual information (MI), recurrence quantification analysis (RQA), and Spearman correlation are the four approaches used to identify pharmacophore features.

The linear association between the various pharmacophore features was determined using ANOVA, and the significant pharmacophore features with the greatest F-values were chosen (Johnson and Synovec, 2002). The F-value is a statistical test used to determine whether the predicted values of a quantitative variable among various pre-defined groups differ from one another. It is determined as the difference between the variances of the sample means and the variances of the individual samples. In our work, the ANOVA technique is applied between each feature and the target vector to obtain the F-value for each feature. The features are then ranked based on their F-value, where the higher the F-value, the more important the feature.

MI (Macedo et al., 2019) is a measure of how much information can be learned about a variable ‘P’ by utilizing a different random variable ‘Q.’ To understand the common information included in all pharmacophore features, the MI value for all pharmacophore descriptors is first calculated. If the MI value is ‘1,’ it is assumed that all the pharmacophore features share the same information, and if it is ‘0,’ it is assumed that none of the features share any common (or special) information, and pharmacophore properties with the greatest MI value were chosen.

In RQA, the measure utilized to rank the pharmacophore properties was entropy, which is the probability distribution of the diagonal line on the RQA plot (Eckmann et al., 1987). It facilitates research on the relationship between the RQA-based entropy measure and the likelihood of discovering probable protein-binding conformations in terms of the time–space evolution of protein conformations. The method contributes to the exploration of the relationship between RQA-based entropy and the likelihood of discovering probable protein-binding conformations.

In the Spearman correlation coefficient (Hauke and Kossowski, 2011), pharmacophore properties are sorted using the absolute value of the correlation coefficient. This approach facilitates the identification of highly correlated pharmacophoric features. The relevant formulas for the four feature selection methods are provided in Supplementary Information.

To identify the important pharmacophore features, a feature ranking score is computed based on the scores obtained from each of the individual methods and the features with the highest ranking score of ‘4’ are retained, indicating that all four ML feature selection approaches identify such a feature as potentially significant for binding.

### 2.5 Validation of pharmacophore models

The capacity of the pharmacophore models to identify the targets’ ligands in ensemble-based docking was assessed using databases of ligands and decoys.

#### 2.5.1 Preparing DUD-E/GDD database—conformation generation

Two widely used publicly accessible datasets were used: i) the Directory of Useful Decoys, Enhanced (DUD-E) (Mysinger et al., 2012) that contains “active” (known ligands) and “decoy” compounds for multiple target receptors including ADORA2 and ADRB2, and ii) the GPCR Decoy Database (GDD) (Gatica and Cavasotto, 2011) that contains “active” and “decoy” molecules for OPRK1 and OPRD1 (Table 2). All active and decoy datasets were included in MOE databases, and their atomic charges were assigned using the MMFF94x force field implemented in MOE. Conformations of “active” and “decoys” were generated using the conformer generation function of the open-source chemistry toolbox Open Babel (O’Boyle et al., 2011). A Python script was developed in-house to process and automate conformer generation, and up to 100 unique conformers for molecules were generated and stored in an MOE database.

**TABLE 2:** Number of active and decoy ligands to each protein.

Protein name	Known ligands (actives)	Decoys
Adenosine receptor A2A	844	10,899
β2-Adrenergic receptor	447	15,255
δ-Type opioid receptor	377	14,703
κ-Type opioid receptor	307	11,973

#### 2.5.2 Pharmacophore search and enrichment calculation

The pharmacophoric features identified, as described in Section 2.4, were used to screen the compounds in the compounds’ databases described above. For every compound in the database, the conformation that showed the minimum RMSD between the pharmacophore query features and their respective ligand annotation points was defined as the best match (Lätti et al., 2016). Although every conformation of a molecule was evaluated against the pharmacophore model, only the best matched conformation was retained and stored in an MOE database, ensuring one representative conformation for each compound which passed the pharmacophore filter.

Following the pharmacophore search, the enrichment factor (EF) was calculated to evaluate the effectiveness of the pharmacophore models in differentiating active ligands from decoys. The EF was calculated using the below equation.

$$EF=\frac{\left(active\;hits\right)\;/\;\left(decoy\;hits\right)\;after\;PH4\;search}{\left(active\;hits\right)\;/\;\left(decoy\;hits\right)\;in\;the\;DUD.E\;or\;GDD\;database}.$$ (1)

#### 2.5.3 Docking and enrichment calculation

The molecules that successfully passed the pharmacophore search were subsequently used for docking on their respective target receptors. The potential binding sites from MOE SiteFinder were used as binding sites for docking. The receptor was treated as rigid, and the binding poses were scored initially by London ΔG scoring, followed by a rescoring using GBVI/WSA ΔG (Labute, 2008). For each compound, the five highest ranked poses based on GBVI/WSA ΔG underwent refinement through molecular mechanics minimization, utilizing the MMFF94x force field followed by calculation of the MM-PBSA interaction energy, as implemented in MOE, between the docked compounds and the protein target. Docking was performed with pharmacophore placement constraint, which allows the binding modes: after the docking refinement stage, only the poses that align with the pharmacophore model are retained. Any docked pose that does not match the pharmacophore query is eliminated. Given that some ligands might be discarded during the docking calculations with pharmacophore placement, the EF was recalculated using Equation 1. Docking jobs were executed either on a local machine using 24 CPU cores or Alabama Supercomputer (ASC) using 60 cores.

**FIGURE 1:**
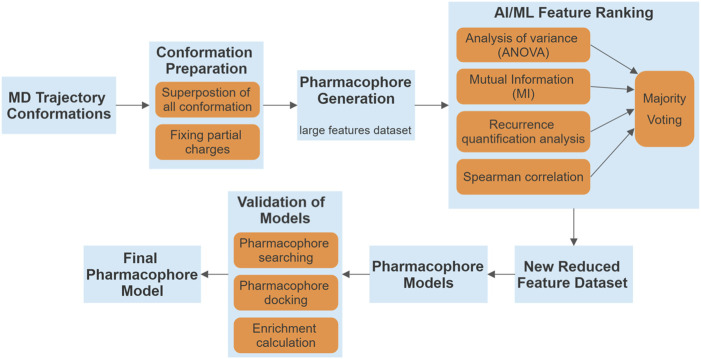
Flowchart illustrating the methodology employed in our study. The process begins with MD trajectory conformations and progresses through several stages, including conformation preparation, pharmacophore generation, and validation of pharmacophore models. AI/ML feature ranking framework integrates various statistical and analytical methods, resulting in a majority voting system to yield a refined set of features.

The flowchart below (Figure 1) illustrates the methodology employed in our study.

### 2.6 Control calculations

Our AL-ML workflow was compared with three pharmacophore-based screening: ligand-based pharmacophores, pharmacophore models generated based on a single crystal structure, and models generated using multiple crystal structures. To make a comparison between our AL-ML method and ligand-based pharmacophore screening, the whole DUD-E or GDD/GLL databases were used, and after superposing all ligands on the co-crystallized ligand as the template, the consensus pharmacophore models were generated with the same number of pharmacophore features, as was achieved in the AL-ML workflow. These models were used to filter the databases of conformers to DUD-E for all four proteins. The AI-ML workflow was also compared to standard X-ray structure-based pharmacophore models, and the same method was followed but by using the co crystalized ligand (of PDB crystal structures in Table 1) in order to generate pharmacophore models. Enrichment was also calculated in the same way. Finally, we generated pharmacophore models following the same method proposed in Figure 1, excluding the AI/ML feature ranking, and using all available PDB crystal structures of the target receptors co-crystallized with unique ligands (Table 3). Pharmacophore features were generated on all prepared crystal structures within a 6.5-Å cutoff from the co-crystallized ligands. These features were then clustered to generate consensus features which summarize the pharmacophore features that are common to multiple structures. Pharmacophore features were sorted by their frequency (Equation 1). Final pharmacophore models were constructed by choosing six to eight features, prioritized based on their frequencies, from the top of the sorted feature list. The enrichment calculations in screening of known active ligands versus decoys were performed similarly as explained (Formula 1). No docking calculations were performed.

**TABLE 3:** Details of sets of known ligands/decoys used.

#	Target receptor	UniProt ID	#PDB crystal	Structures with a unique ligand	GDD entry
1	Adenosine receptor A2A (ADORA2A)	P29274	58	24	AA2AR_HUMAN
2	β2-Adrenergic receptor (ADRB2)	P07550	39	17	ADRB2_HUMAN
3	δ-Type opioid receptor (OPRD1)	P41143	5	5	OPRD_HUMAN
4	κ-Type opioid receptor (OPRK1)	P41145	5	5	OPRK_HUMAN

## 3 Results

### 3.1 Pharmacophore models generated using all available MD conformations

**FIGURE 2:**
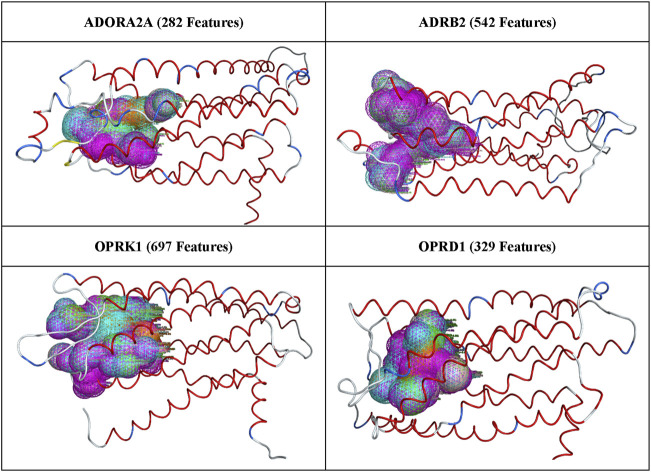
All pharmacophore features generated for each protein receptor.

After superposition of all 3,000 available conformations from MD trajectories for each of the four proteins of interest, the pharmacophore features generated, as described in Methods, are illustrated in Figure 2.

The number of pharmacophoric features listed in Figure 2 varies between ∼300 and ∼700, indicating that most binding site pharmacophores in a protein trajectory were present in several of the protein conformations. These pharmacophore features were run through the AI/ML workflow described above, and the output features were combined to create the final pharmacophore models, which were then selected for validation through pharmacophore search and docking, as described in Methods.

### 3.2 AI/ML framework

The number of pharmacophoric features identified as ‘important” for each of the four proteins is given in Table 5, line “no threshold”, i.e., including all pharmacophore features in ML processing.

In addition, we also used fractions of the pharmacophoric features that are not widely represented in the entire trajectory. The rationale behind that is that since the number of protein conformations selected by the ligand is a fraction of the total number of conformations sampled by the protein, the pharmacophore features associated with such conformations may be found less frequent but still passing the ML criteria and defined as ‘important.’ In other words, pharmacophore features found in most of the conformations may not be relevant for binding and may lead to oversampling.

We have, hence, used the ML approaches described above with only pharmacophore features present in up to 5%, 10%, 15%, 20%, or 25% of the total number of pharmacophores. The corresponding number of pharmacophores for each of the four proteins is given in Table 4, and the number of such pharmacophores passing the ML criteria is given in Table 5.

**TABLE 4:** Number of pharmacophore features after applying thresholds of frequencies.

Frequency threshold range	# of pharmacophore features for each protein			
	ADORA2A	ADRB2	OPRK1	OPRD1
No threshold	282	542	697	329
Between 1% and 5%	108	153	279	99
Between 1% and 10%	125	183	342	119
Between 1% and 15%	134	201	368	128
Between 1% and 20%	143	213	382	141
Between 1% and 25%	148	223	388	146

**TABLE 5:** Important pharmacophore features selected using the AI/ML framework.

Frequency threshold range	# of important pharmacophore features selected using the AI/ML framework for each protein			
	ADORA2A	ADRB2	OPRK1	OPRD1
No threshold	26	6	19	33
Between 1% and 5%	6	0	4	8
Between 1% and 10%	8	2	7	9
Between 1% and 15%	10	5	7	14
Between 1% and 20%	16	5	8	14
Between 1% and 25%	20	7	7	19

### 3.3 Pharmacophore search and docking

The pharmacophore features listed in Table 5 were used as a filter for the compound databases, as described in Methods, i.e., with pharmacophore screening only along with pharmacophore-directed docking.

Table 6 shows the maximum enrichment by pharmacophore search achieved from each threshold range, and these results are summarized in Table 7. The overall enrichment varies from one protein to the other from a relatively mediocre 5.2 to a very high 54.3 The cloud of pharmacophoric features shown in Figure 2 is very large, in hundreds. For ADRB2, 6 out of 542 features selected using the AI/ML framework on a dataset with no threshold provided the best results in terms of enrichments, while in case of ADORA2A, limiting the data between a frequency threshold of 1% and 10% provided the highest enrichment. In the case of OPRK1, the best enrichment from the full 697 pharmacophores is found when processing pharmacophores present in up to 25% to achieve the highest enrichment ratio. In some cases, shown with NA in Table 6 below, we could not obtain any enrichment (either 0 actives or 0 compounds passed the pharmacophore filter). In the case of OPRD1, no clear results could be obtained without selecting manually seven pharmacophore features from the “no threshold” 34 features, suggesting a possible imbalance ratio of binding vs. non-binding conformations in the OPRD1 MD trajectory.

**TABLE 6:** Maximum enrichments in case of each protein.

	Protein			
	ADORA2A	ADRB2	OPRD1	OPRK1
Total number of features in the cloud of pharmacophores	282	542	329	697
Frequency threshold range	Max enrichment			
No threshold (original)	2.4	**54.2**	**5.2**	NA
Threshold between 1% and 5%	0.9	NA	NA	1.2
Threshold between 1% and 10%	**14.9**	NA	NA	3.9
Threshold between 1% and 15%	NA	13.7	NA	3.9
Threshold between 1% and 20%	NA	9.8	NA	0.0
Threshold between 1% and 25%	NA	3.3	NA	**8.1**

Maximum enrichments are shown in bold.

**TABLE 7:** Enrichment of pharmacophore models.

Protein	Enrichment Models by MD trajectories	
	Step 1 Pharmacophore search	Step 2 Pharmacophore dock
ADORA2A	14.25	14.90
ADRB2	15.93	54.27
OPRD1	4.78	5.26
OPRK1	5.57	8.12

The comparison between our AI-ML approach and ligand-based pharmacophore models is summarized in Table 8, which shows a significant improvement in using the AI-ML platform in case of ADORA2A, ADRB2, and OPRK1, while a slight improvement in case of OPRD1 receptors. The comparison between our AI-ML approach and X-ray structure-based pharmacophore models, as shown in Table 9, showed improvement in screening actives versus decoys using our AI-ML workflow. Although in case of ADRB2, we observed a slight decrease in enrichment numbers, although both methods provided significant enrichment.

**TABLE 8:** Comparison between the AI-ML model and ligand-based pharmacophore models.

Protein	Enrichment based on ligand-based pharmacophore models	Enrichment based on the AL-ML workflow
ADORA2A	5.10	14.90
ADRB2	3.04	54.27
OPRD1	4.88	5.26
OPRK1	1.97	8.12

**TABLE 9:** Comparison between the AI-ML model and X-ray structure-based pharmacophore models.

Protein	Enrichment based on X-ray structure-based pharmacophore models	Enrichment based on the AL-ML workflow
ADORA2A	3.3	14.90
ADRB2	63.73	54.27
OPRD1	3.47	5.26
OPRK1	3.57	8.12

**FIGURE 3:**
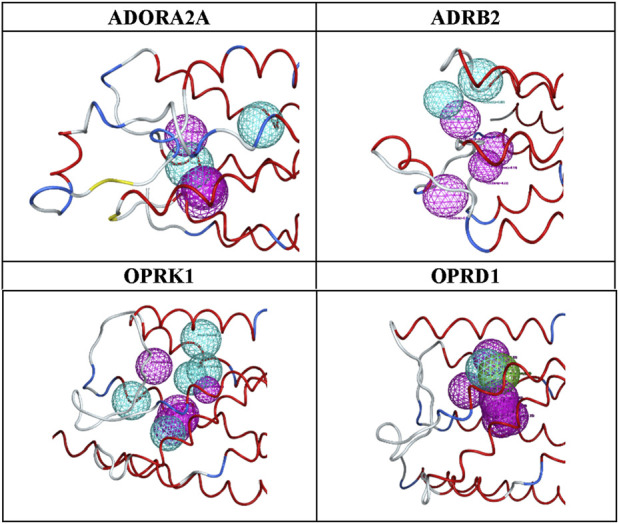
Final pharmacophore models.

Additional research is necessary to more effectively and systematically handle the vast amount of available data, eliminate data redundancy and noise, and enhance the enrichment ratios. Potential strategies could include more fine-grained and bins of the number of pharmacophore features processed by ML, or clustering MD conformations prior to their use in this ensemble pharmacophore generation workflow, rather than using the whole MD trajectories, to limit potential data imbalance. The output of AI/ML feature ranking is shown in Table 10 and Figure 3.

**TABLE 10:** Selected PH4s from the AI/ML feature ranking framework.

ADRB2	ADORA2A	OPRD1	OPRK1
F1_Don	F1_Don	1_Don	F1_Acc
F2_Acc	F2_Don	F2_Don	F2_Don|Acc
F3_Acc	F3_Don	F3_Don	F3_Acc
F4_Don	F4_Aro|PiR	F4_Acc	F4_Acc
F5_Don	F5_Acc	F5_Don	F5_Acc
F6_Don	F6_Aro|PiR	F6_Don|Acc	F6_Acc
	F7_Don	F7_Hyd|HydA	F7_Acc
	F8_Don		

**FIGURE 4:**
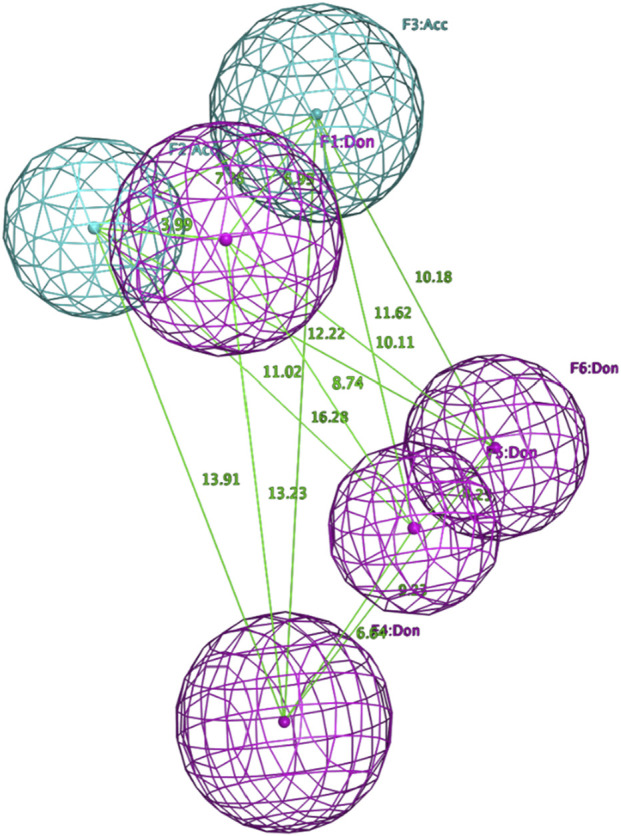
Final pharmacophore model of ADRB2.

**FIGURE 5:**
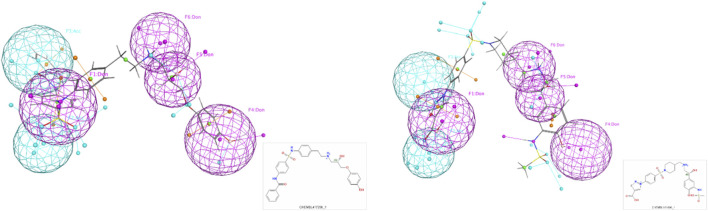
Examples of the top scoring ligand in the final pharmacophore model.

In addition, a detailed example of one of these models with top scoring active ligands matching the pharmacophore features is shown in Figures 4, 5.

The advantages of the results shown in Figure 3 are clear: a limited number of pharmacophores, leading to an enrichment of databases in ligands versus decoys, which can be easily interpreted in terms of chemical and structural features. As shown in Figure 3 and Table 10, the number and locations of the pharmacophores are not identical for each of the four proteins. On one hand, this is not unexpected since the GPCR proteins used here have their own set of ligands. However, on the other hand, it is not possible to assertively assess, at this early stage of the research, how many other possible combinations of pharmacophores could be identified.

## 4 Discussion/conclusion

The work presented here is a first step toward using ML to process an ocean of structural and chemical protein properties to be used in ML in a way that makes the ML analysis easy, intuitive, and actionable for lead optimization. In Gupta et al. (2022a), we used feature scoring to identify unique descriptors that can aid in distinguishing between binding and non-binding protein descriptors, whereas in this work, we use the unique pharmacophores to be better able to identify actives from a dataset that consists of both actives and decoys. The aim of the AI/ML approach is to make the dataset less complex in both situations so that instead of requiring a supercomputer, we can perform all the computations locally. Table 11 shows the comparison of our current results to our previous (Gupta et al., 2022b) findings, where the enrichment ratio values were computed to validate the ML protein conformation selection/prediction framework. The results proposed here show that this is a possible goal that such medicinal chemistry-friendly properties lend themselves well to the kind of ML processing that is usually efficient for purely numerical—and very arcane—“haystacks of numbers.” Yet much more work is needed for 1) identifying the optimum ways to combine the pharmacophores and 2) characterize the specificity of not only the protein conformations selected by their ligands but also how the selected conformations differ between them. The ultimate goal, *in fine*, will be to identify—if they exist—the structural and chemical features that will lead to ligand binding of the trajectory of a novel protein, about which no or very little binding data are already known. This is a difficult, maybe distant, goal, but the road to success will undoubtedly require significant ML-based data analysis, justifying the efforts the community invests in ML and AI, as illustrated in this special issue of *Frontiers in Molecular Biosciences*.

**TABLE 11:** Comparison between our current results and previous calculations.

Protein	Previous enrichment (Gupta et al. 202b)	Current enrichment
ADORA2A	∼10–12.5	14.9
ADRB2	∼18–24	54.27
OPRD1	∼10–37	5.26
OPRK1	∼13–27	8.12

## Data Availability

The raw data supporting the conclusions of this article will be made available by the authors, without undue reservation.
