# Supplementary material for: Linking machine learning and biophysical structural features in drug discovery
Source: Front Mol Biosci. 2025 Jan 23;11:1305272. doi: 10.3389/fmolb.2024.1305272 (PMC11798802; doi:10.3389/fmolb.2024.1305272)
Supplement: Supplementary file 1 [file DataSheet1.docx]

# Supplementary information

**Relevant formulas**

Following are the formulas used in ANOVA, MI, RQA and spearman correlation calculations. These equations have been published in our previous papers.

**Analysis of Variance (ANOVA)**

The value calculated by the ANOVA equations is an ‘‘f value of the class-to-class variance over the within-class variance. The between class variance is calculated as:

$\sigma_{between-class}^{2}= \frac{\Sigma{(\overline{a_{i}}- \overline{a})m_{i}}^{2}}{(s - 1)}$                                                    (1)

where mi is the number of measurements in the i^th^ class, ai is the mean of the i^th^ class, and a is the overall mean. The within class variance is calculated as:

$\sigma_{within-class}^{2}= \frac{(\Sigma\Sigma{(a_{ij}- \overline{a})}^{2}) - (\Sigma{(\overline{a_{i}}- \overline{a}))m_{i}}^{2})}{(N - s)}$ (2)

Where aij is the ith measurement of the jth class. An ANOVA f ratio is then calculated as the ratio between the two variances:

$f$value = $\frac{\sigma_{between-class}^{2}}{\sigma_{within-class}^{2}}$                                  (3)

**Mutual Information**

Given a variable **Y** and the conditional entropy **H**(**A**|**B**) of **A** with respect to **B** is defined as:

**H**(**A**|**B**) = $- \sum_{b\in B} \sum_{a\in A} \sum_{a\in A} p(a,b) log(p(a|b))$ (4)

**H(A)** = $-\sum_{a\in A} p(a) log(p(a))$ (5)

Where,

- p(a,b) is the joint probability density function.
- p(a|b) is the posterior probabilities of **A** given **B**.
- p(a) is the probability density function

From equation (4) and (5), mutual information **I**(**A**;**B**) can be defined as below:

**I**(**A**;**B**) = **H**(**A**) - **H**(**A**|**B**) = $-\sum_{b\in B} \sum_{a\in A} p(a,b)log\frac{p(a,b)}{p(a) p(b)}$ (6)

In equation (6), if the MI value **I** is 1, then **A** and **B** are dependent on each other, i.e., protein features share similar information. If the MI value **I** is 0, then **A** and **B** are independent of each other i.e., no common (in other words unique) information between the features.

**Recurrence Quantification Analysis**

One of the variables generated by the quantification of the recurrences is Entropy (ENT), which is the probability distribution p(j) of the diagonal line on the RQA plot and is defined as:

$ENTR = -\sum_{i = i_{min}}^{N} p(i) ln(p(i))$                     (7)

where N is the number of points on the state space trajectory and i is the length of the diagonal line in the RQA plot.

**Spearman Correlation Coefficient**

The correlation coefficient for each feature is obtained by applying the formula as defined below:

$\rho= \frac{\Sigma_{i}\left( a_{i}-a \right)\left( b_{i}-\underline{b} \right)}{\sqrt{\sum_{i} \left( a_{i}-\overline{a} \right)^{2} \left( b_{i}-\overline{b} \right)^{2}}}$               (8)

where a is the feature vector and a is its corresponding mean. Similarly, b is the target vector and b is the mean of the target vector.
